# Impact of Response Assessment Intervals on Survival and Economic Burden in Long-Term Responders to Immunotherapy for Advanced Non-Small-Cell Lung Cancer

**DOI:** 10.3390/cancers17203312

**Published:** 2025-10-14

**Authors:** Min Wang, Vannhong Soth, Xingzhu Liu, Yuxi Li, Xianyan Chen, Jianxin Xue, Youling Gong

**Affiliations:** 1 Division of Thoracic Tumor Multidisciplinary Treatment, Cancer Center and State Key Laboratory of Biotherapy, West China Hospital, Sichuan University, Chengdu 610041, China; 2 Division of Abdominal Tumor Multimodality Treatment, Cancer Center, West China Hospital, Sichuan University, Chengdu 610041, China

**Keywords:** immunotherapy, long responders, response assessment, non-small-cell lung cancer, economic burden

## Abstract

**Simple Summary:**

Immunotherapy has emerged as a breakthrough for the treatment of advanced non-small-cell lung cancer (NSCLC), significantly improving patient survival. However, it has also led to an increase in the costs associated with response assessment. This study supports that extending the response assessment interval from 2 to 3 months maintains overall survival without compromising advanced NSCLC long-term responders to immunotherapy. This approach using less frequent monitoring may substantially reduce annual healthcare expenditure in the US by nearly USD 6 million.

**Abstract:**

Background: Immunotherapy has emerged as a breakthrough for the treatment of advanced non-small-cell lung cancer (NSCLC), significantly improving patients’ progression-free survival (PFS) and overall survival (OS). However, the medical burden of response assessment has worsened for long-term maintenance therapy. It remains unclear whether a specific response assessment interval could provide both survival benefits and cost savings. Methods: We retrospectively included patients with advanced NSCLC who underwent immunotherapy and achieved PFS > 12 months. We utilized propensity score matching (PSM) to reduce the selection bias. The survival outcomes were evaluated using the log-rank test and Cox proportional hazard models, while the economic impact was assessed through the performance of a cost minimization analysis (CMA). A medical expenditure extrapolation model was developed based on epidemiological statistics and data from clinical trials. Results: After PSM, a total of 376 patients were included. The survival difference was not significant [hazard ratio (HR) = 0.78, 95% confidence intervals (CIs) = 0.53–1.14; *p* = 0.200] between the 2-month response assessment group (*n* = 188) and the 3-month response assessment group (*n* = 188). Patients receiving immunotherapy alone and those with a positive PD-L1 expression experienced a significant survival benefit. Our extrapolation model projects that, annually, there will be approximately 7026 new long-term responders to immunotherapy in the United States. Adopting a 3-month assessment strategy could reduce annual healthcare expenditure by nearly USD 6 million. Conclusions: This study presented the first statistical evidence supporting a refined response assessment strategy for long-term responders to immunotherapy with advanced NSCLC. These findings support the adoption of a less frequent, yet equally effective, monitoring approach to make tumor surveillance more precise and cost-effective.

## 1. Introduction

Lung cancer is the most common cancer and the leading cause of death from cancer globally [1]. Non-small-cell lung cancer (NSCLC) is the most common type of lung cancer; it accounts for about 85% of lung cancers [2], with nearly 70% of patients presenting with locally advanced or metastatic disease at diagnosis [3,4].

Regarding advanced NSCLC, a large number of randomized clinical trials have confirmed immunotherapy to be a breakthrough and a new standard of care, substantially extending both progression-free survival (PFS) and overall survival (OS) [5,6,7,8]. Immune checkpoint inhibitors (ICIs) mainly targeting programmed death-1 (PD-1)/programmed death ligand 1 (PD-L1) activate antitumor immunity and provide a durable survival benefit [9], particularly in PD-L1-high NSCLC [10]. The KEYNOTE-024 [11] trial reported that 25.8% (39/151) advanced cancer patients received 35 cycles of pembrolizumab monotherapy for 2 years. Among these 39 patients, 32 were still alive 5 years after random assignment. Similarly, in trials investigating the administration of pembrolizumab plus chemotherapy, KEYNOTE-189 [12] and KEYNOTE-407 [13] indicated that more than one-tenth of patients (13.9% and 19.8%, respectively) underwent the 2-year maintenance therapy. For these patients, the 3-year OS rates from the time of completing therapy were 71.9% and 69.5%, respectively.

However, while immunotherapy greatly improves patients’ PFS and OS, it imposes a significant financial burden on patients and healthcare systems. An Italian study reported an average financial burden of EUR 25,859 per NSCLC patient over 16.4 months of follow-up [14]. Although pharmacological costs still account for the majority of expenses, the high frequency of medical visits and response assessment expenditure should also be of concern. The National Comprehensive Cancer Network (NCCN) [15] guidelines recommended that patients with advanced NSCLC should undergo response assessments every 6 to 12 weeks during maintenance therapy. In the clinical trial setting, patients are typically assessed every two cycles (42 ± 7 days). In routine clinical practice, however, the frequency is more variable, and assessments may occur every two to three cycles. A recent study suggested that reduced surveillance for metastases could benefit patients who have received radical treatment [16]; it highlighted that the potential harms of surveillance included not only the financial costs but also the surveillance-related anxiety, reduction in asymptomatic time during which quality of life could be higher, and radiation exposure. However, for advanced-stage patients who have achieved long-term survival with a sustained response, optimal surveillance strategies remain undefined due to a lack of evidence.

To the best of our knowledge, no studies have yet systematically elucidated the impact of response assessment intervals on survival and economic burden for long-term responders to immunotherapy who have advanced NSCLC. This retrospective study aimed to determine the applicable assessment interval to facilitate clinical practice and consider the economic benefits concerned.

## 2. Materials and Methods

### 2.1. Target Population

Our study cohort was derived from West China Hospital, Sichuan University, from 2018 to 2023. The inclusion criteria were as follows: age ≥ 18 years; pathologically confirmed NSCLC; AJCC 8th edition stage IV; underwent first-line immunotherapy and maintenance therapy; Eastern Cooperative Oncology Group Performance Status (ECOG-PS) 0–1; PFS > 12 months. Patients were excluded if they lacked accessible assessment records within our hospital’s Health Information System (HIS) or from external facilities where they underwent subsequent examinations. The study protocol was approved by the institutional research ethics committee of West China Hospital of Sichuan University, and the need for written informed consent was waived because the data were retrospective and unidentifiable.

### 2.2. Collection of Variables

We collected patient demographics (age, gender, smoking history), histological type, PD-L1 expression (<1%, 1–49%, ≥50%), ECOG-PS, immunotherapy drugs, immunotherapy monotherapy (yes/no), radiotherapy history (yes/no), as well as all medical imaging, laboratory tests, and response assessment records. Tumor response evaluation was measured by The Response Evaluation Criteria In Solid Tumors (RECIST Version 1.1) by two oncologists. The primary endpoint was OS, defined as the time from diagnosis to death, or the follow-up deadline (30 April 2024).

### 2.3. Classification of Response Assessment Strategy

In clinical practice, there is variability in imaging intervals due to scheduling conflicts from holidays, treatment-related interruptions, and personal reasons. Therefore, we established a dual-metric validation to classify the response assessment group using the median value (9 weeks) of 6–12 weeks as the stratification threshold. This approach combined the (1) mean interval (total observation duration divided by number of imaging assessment scans) and (2) median scan interval. Successful group classification occurred when both of the metrics aligned. For discordant cases, we manually reviewed the patient’s clinical records to identify the reasons for discrepancies. Cases with unexplainable deviations were excluded from this study.

### 2.4. PFS Stratification

Methodologically, patients were stratified into two groups based on PFS, using the median value of 21.08 months as the cutoff. This stratification was necessary because, in clinical practice, patients with longer PFS often undergo a gradual reduction in the frequency of their response assessments. This practice naturally leads to a larger proportion of long-PFS patients being followed-up at 3-month intervals, creating a significant imbalance between the comparison groups. To mitigate this confounding effect, we employed both PFS stratification and propensity score matching (PSM).

### 2.5. Economic Analysis and Modeling Process

CMA was used for economic evaluation. Based on the World Health Organization’s (WHO) epidemiologic statistics and data from the KEYNOTE-042 [8], 189 [12], and 407 [13] clinical trials, an extrapolation model was developed. The modeling process was as follows: NSCLC constitutes approximately 85% of all lung cancer cases [17], with 34.4% of these being advanced cases [18]. After excluding patients eligible for targeted therapy (*EGFR mutation*: 24.4% [19]; *ALK*: 4.4% [20]; *ROS1 fusion*: 2.59% [21]), approximately 68.2% of the advanced patients were estimated to be candidates for immunotherapy. According to our study, the distribution of first-line immunotherapy regimens was as follows: 38.8% received immunotherapy monotherapy, 46.4% received chemoimmunotherapy for non-squamous NSCLC, and 14.8% received chemoimmunotherapy for squamous NSCLC. Based on 5-year data from KEYNOTE-042 (immunotherapy monotherapy), KEYNOTE-189 (non-squamous chemoimmunotherapy), and KEYNOTE-407 (squamous chemoimmunotherapy), 16.0%, 13.9%, and 19.8% of patients, respectively, completed 35 cycles of maintenance immunotherapy (PFS > 24 months). Using this model, the proportion of patients receiving immunotherapy who achieve long-term responses can be estimated.

The NCCN guidelines for the management of immune-related adverse events (irAEs) [22] recommended that patients undergoing immunotherapy should receive complete blood count (CBC) and comprehensive metabolic panel (CMP), prior to each treatment or every 4 weeks during immunotherapy, and thyroid-stimulating hormone (TSH) and free thyroxine (FT4) every 4 to 6 weeks during immunotherapy. CT with or without contrast every 6 to 12 weeks was also suggested during maintenance therapy for those with known or high-risk sites of disease. Based on these recommendations, we defined the following tests as essential for response assessment in our extrapolation model: Chest CT (with and without contrast), Abdomen CT (with and without contrast), CBC, CMP, TSH, and FT4.

U.S. News has ranked the best cancer hospitals for treatment, and we evaluated the price transparency files of the Top 10. Hospital standard charges are often much higher than the actual payments, and these charges vary for each individual. Significant variations arise from a patient’s specific medical condition, care setting, and the negotiated rates established with their health insurance company. Therefore, our analysis employs the minimum standard charges (USD) from the price transparency files as the base cost (obtained from these hospitals’ websites). We then calculated the mean price across hospitals as the final estimated price for each test.

### 2.6. Statistical Analysis

Survival benefits were evaluated using Kaplan–Meier estimators (median overall survival [mOS] in months with 95% confidence intervals [CIs] was derived) and Cox proportional hazard models. Categorical variables were compared using Pearson’s chi-square test. Continuous variables were compared using Student’s *t*-test or the Mann–Whitney U test, as appropriate. All analyses were performed using R (version 4.3.2), and all statistical tests were two-sided with a significance threshold of *p* < 0.05. To control for potential confounding factors due to imbalances in the baseline characteristics, PSM was performed using the “MatchIt” package in R. Factors were matched in a 1:1 ratio with a caliper width of 0.1.

## 3. Results

### 3.1. Patient Features

In this retrospective study, 7942 immunotherapy cases were retrieved from the database, and 588 NSCLC cases were selected for the PSM. The cohort consisted of 476 (81.0%) males and 112 (19.0%) females, with a median age of 63 years. Most patients (71.9%) had non-squamous cell carcinoma, while 28.1% had squamous cell carcinoma. A total of 229 patients followed a 2-month response assessment strategy, and 359 followed a 3-month strategy. The median follow-up was 28.7 [95% CI: 27.0–32.7] months. In total, 177 deaths were recorded.

**Figure 1:**
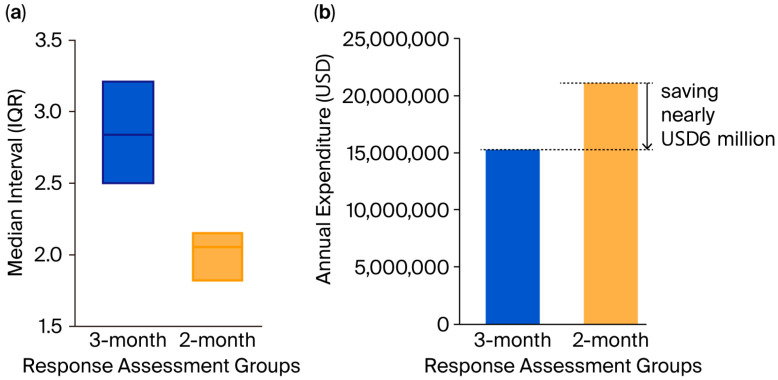
Histograms. (**a**) In the 2-month response assessment group, the median interval was 2.05 months (IQR: 1.81–2.17), whereas in the 3-month group (*n* = 188), it was 2.84 months (IQR: 2.50–3.22). (**b**) Estimated annual expenditure histogram for the two response assessment groups: compared with the 2-month strategy, 3-month intervals reduced annual response assessment expenditure by a conservative estimate of USD 5,883,310.34 (2-month group: USD 21,150,128.32 vs. 3-month group: USD 15,266,817.97).

Before the PSM, significant differences existed in terms of PD-L1 expression, PFS stratification, immunotherapy drugs, and radiation history. The baseline characteristics of the patients before the PSM are detailed in Table 1. After the PSM, the analysis yielded a well-balanced final cohort of 376 patients (188 in each group; Table 1). The median interval of response assessments in the 2-month group (*n* = 188) was 2.05 months (interquartile range [IQR]: 1.81–2.17), whereas in the 3-month group (*n* = 188), it was 2.84 months (IQR: 2.50–3.22, Figure 1a).

### 3.2. Survival Analysis

**Figure 2:**
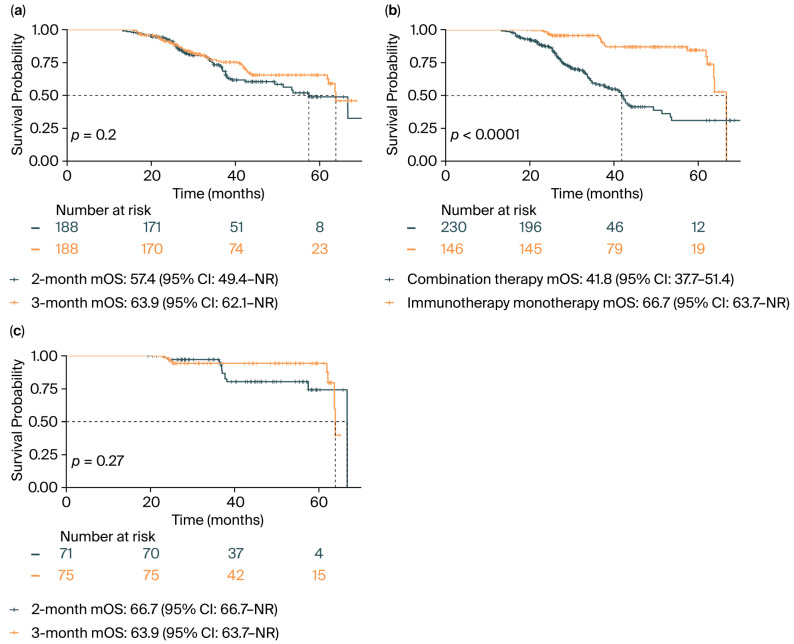
Kaplan–Meier curves: (**a**) The Kaplan–Meier curve of the two response assessment groups showed that there was no significant difference between the 2-month and 3-month groups. (**b**) The Kaplan–Meier curve of different therapy groups showed a significant survival benefit for patients receiving immunotherapy monotherapy compared with those in the combination therapy group. (**c**) The Kaplan–Meier curve of the immunotherapy monotherapy subgroup showed that there was no significant difference between the 2-month and 3-month groups.

As shown in Figure 2a, there was no significant difference in overall survival between the two groups (mOS: 57.4 months [95% CI: 49.4–not reached (NR)] for the 2-month group vs. 63.9 months [95% CI: 62.1-NR] for the 3-month group; *p* = 0.200). Consistent with this, the Cox regression analysis revealed a nonsignificant hazard ratio (HR) of 0.78 [95% CI: 0.53–1.14] for the 3-month group (Table 2).

In the multivariate analysis, age ≥ 63 years was significantly associated with a higher risk of death (HR: 2.03 [95% CI: 1.35–3.06]; *p* = 0.001). In contrast, patients who received immunotherapy monotherapy had a significantly lower HR (0.24 [95% CI: 0.14–0.40]; *p* < 0.001). Significant survival benefits were also observed in patients with positive PD-L1 expression compared with those with <1% expression (for expression ≥ 50%: HR: 0.26 [95% CI: 0.10–0.69]; *p* = 0.007). The multivariate model also included gender, smoking history, histological type, radiotherapy history, and immunotherapy drugs; however, none of these variables showed a statistically significant association with overall survival.

We found some significant baseline differences between the immunotherapy monotherapy and combination therapy groups (Table S2). Patients receiving immunotherapy monotherapy were significantly more likely to be ≥63 years old (56.2% vs. 38.3%), have PFS exceeding the median (65.8% vs. 42.2%), and exhibit PD-L1 expression ≥ 50% (43.8% vs. 30.9%). No significant differences were observed in terms of gender distribution, histological type, smoking history, radiotherapy history, or response assessment strategies between groups (*p* > 0.05). The Kaplan–Meier curve (Figure 2b) showed a significant survival benefit for patients receiving immunotherapy monotherapy (mOS: 66.7 [95% CI: 63.7-NR]) compared with those in the combination therapy group (mOS: 41.8 [95% CI: 37.7–51.4]; *p* < 0.001). Among patients receiving immunotherapy monotherapy (Figure 2c), no statistically significant difference in overall survival was observed between the 2-month and 3-month response assessment groups (2-month group mOS: 66.7 [95% CI: 66.7-NR] vs. 3-month group mOS: 63.9 [95% CI: 63.7-NR]; *p* = 0.270).

### 3.3. Economic Cost Analysis and the Extrapolation Model

**Figure 3:**
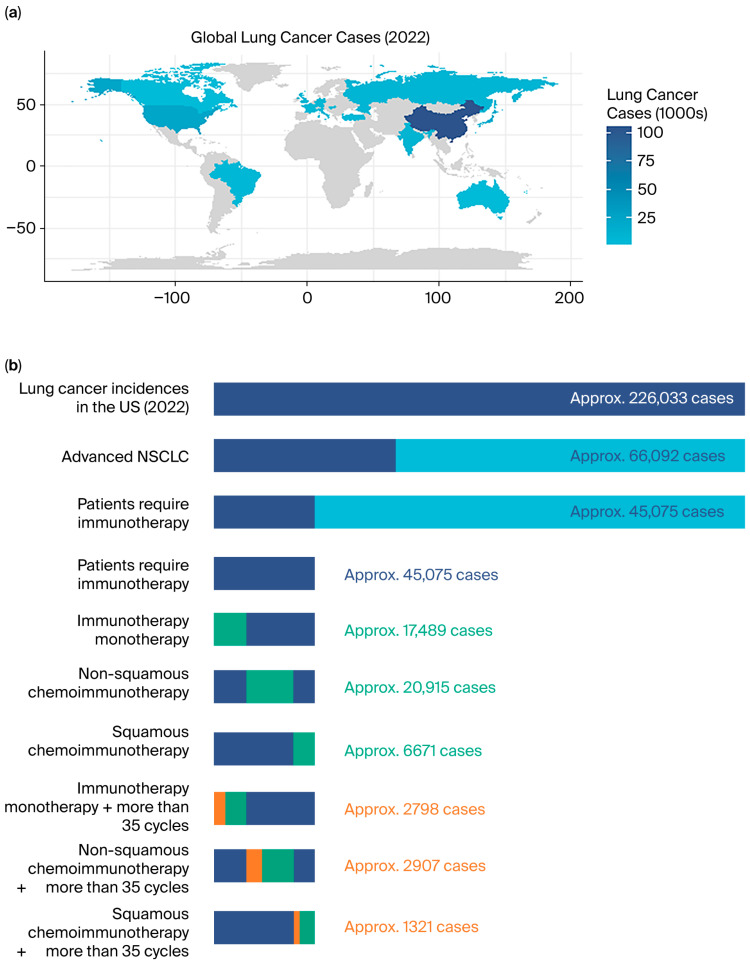
The extrapolation model flowchart. (**a**) The map of the WHO epidemiologic statistics for global lung cancer cases in 2022. Based on the data, we estimated that there were 226,033 lung cancer cases in the United States. (**b**) Based on the WHO epidemiologic statistics and the clinical trials’ data, an extrapolation model was developed. Non-small-cell lung cancer (NSCLC) accounts for approximately 85% of all lung cancer cases, with 34.4% advanced cases. After excluding patients who were eligible for targeted therapy, approximately 68.2% of advanced patients were candidates for immunotherapy (according to our study, the distribution of first-line immunotherapy regimens was as follows: 38.8% received immunotherapy monotherapy, 46.4% received chemoimmunotherapy for non-squamous NSCLC, and 14.8% received chemoimmunotherapy for squamous NSCLC). Based on 5-year data from KEYNOTE-042 (immunotherapy monotherapy), KEYNOTE-189 (non-squamous chemoimmunotherapy), and KEYNOTE-407 (squamous chemoimmunotherapy), 16.0%, 13.9%, and 19.8% of patients, respectively, completed 35 cycles of maintenance immunotherapy (PFS > 24 months). Finally, based on the estimated 226,033 lung cancer cases in the United States, we estimated that there will be approximately 7026 new long-term responders to immunotherapy.

Based on the estimated 226,033 lung cancer cases in the United States (2022), we estimated that there would approximately be 7026 new long-term responders to immunotherapy (defined as PFS > 24 months after 35 immunotherapy cycles). The extrapolation model flowchart is shown in Figure 3. Details from five hospitals, where both minimum and maximum standard charge ranges were available, are summarized in Table 3. The minimum standard charge for a single response assessment was USD 514.24, and the maximum standard charge was USD 7073.79. If, for these new cases, the 3-month strategy was followed, this could reduce annual response assessment expenditure by a conservative estimate of USD 5,883,310.34 (2-month group: USD 21,150,128.32 vs. 3-month group: USD 15,266,817.97, Figure 1b), without compromising patients’ overall survival. This suggests that the less-intensive response assessment represents a cost-minimization strategy relative to the more intensive one. Moreover, it is important to note that since we used the minimum standard charges, our model may underestimate the actual cost savings.

## 4. Discussion

This retrospective study evaluated the impact of response assessment intervals on survival and economic burden in advanced NSCLC patients with long PFS following immunotherapy. For the first time, with a statistically rigorous analysis, we revealed a more rational response assessment interval for long-term responders to improve health economics. Furthermore, an extrapolation model was developed to evaluate the potential economic benefits within the US healthcare system.

Tumor response assessments are pivotal in the tumor treatment process. The NCCN guidelines recommended that patients receive a response assessment every 6–12 weeks during their maintenance [15]; however, there is a lack of evidence regarding the optimal interval for survival outcomes and associated economic implications. Our study compared the two most common response assessment strategies in clinical practice: the 2-month strategy and the 3-month strategy. No significant difference in overall survival was observed between these two groups; however, the annual costs were lower in the 3-month group compared with those in the 2-month group. We suggest that, for advanced NSCLC patients receiving immunotherapy with PFS exceeding 12 months, clinicians may consider extending these patients’ response assessment intervals, thus alleviating the financial burden without compromising survival outcomes.

While immunotherapy enables durable remission in advanced NSCLC, the molecular mechanisms underlying sustained response remain incompletely characterized. A recent study by Joan et al. [23] identified that a unique immune microenvironment profile, characterized by high tumor mutational burden (TMB)/PD-L1 expression and low burden of somatic copy number alterations (SCNAs), can predict long PFS (>18 months). This provides a mechanistic basis for disease stabilization in sustained responders. Notably, our study also exhibited a higher survival benefit for PD-L1-positive patients. For specific favorable subgroups, such as PD-L1-positive patients or those receiving immunotherapy monotherapy, a precision-guided extension of assessment intervals may be cost-effective.

Response assessment primarily integrates clinical evaluation (history and physical examination), laboratory tests, and tumor burden quantification via medical imaging. Laboratory tests facilitate the critical surveillance of irAEs. According to the American Society of Clinical Oncology (ASCO) and NCCN guidelines [22], the testing interval for thyroid abnormalities is 4–6 weeks. For myocarditis, the most life-threatening irAE [24,25], the baseline myocardial assessment is performed one week after the first ICI and, if normal, is followed by a 3-month cycle guided by clinical symptoms and electrocardiograms. While this comprehensive monitoring protocol effectively optimizes irAE management, current evidence lacks rigorous cost–benefit analyses of such surveillance strategies. A strict cardiac reassessment strategy for high-risk patients is considered necessary due to its safety implications. However, the clinical necessity of 4–6-week thyroid monitoring may warrant reconsideration, as most abnormalities (e.g., subclinical hypothyroidism) are relatively controllable and seldom mandate treatment cessation. Additionally, imaging monitoring is essential for response assessment; however, its radiation-related harm requires careful risk–benefit evaluation. A recent multinational research [26] demonstrated a significant dose–response relationship between CT radiation exposure and glioma incidence. Furthermore, cumulative CT radiation has been associated with elevated risks of multiple malignancies, including skin cancer, breast cancer, and hematologic neoplasms [27,28]. Epidemiological evidence has indicated that two to three standard CT scans (30–90 mSv) can significantly increase the risk of radiation-induced cancer [29,30]. Therefore, clinical imaging strategies should be personalized to minimize exposure to radiation while maintaining diagnostic accuracy.

The present study investigated the correlation between response assessment strategies and survival outcomes in long-PFS patients receiving immunotherapy while simultaneously conducting a pioneering economic analysis of these strategies’ associated burden. However, several limitations of this study must be acknowledged. Firstly, based on its retrospective nature, observational bias was unavoidable when interpreting these results. The absence of patient-reported utility values and quality-adjusted life years (QALYs) precluded a cost–utility analysis (CUA), which is the preferred pharmaco-economic method for evaluating interventions that exhibit substantial differences in terms of both clinical benefit and cost between treatment groups. Instead, we employed CMA due to the absence of significant differences in OS and the unavailability of utility data. Secondly, although clinical guidelines recommend more frequent laboratory testing than imaging, our study considered one laboratory test plus one imaging test as a single response assessment event. This approach may have led to an underestimation of potential cost savings. Furthermore, only direct medical costs were included, omitting direct non-medical costs (e.g., transportation) and indirect economic burdens (e.g., productivity loss) [31,32]. This also resulted in an underestimation of the annual expenditure. Moreover, the model was based on data from the U.S. healthcare system, and the variation in response assessment costs across hospitals and regions may limit the generalizability of our findings. Finally, the limited sample size and the absence of an external validation cohort may have introduced selection and information bias. To enhance the reliability of future findings, prospective multicenter studies with larger cohorts and independent validation datasets are warranted.

## 5. Conclusions

This study presented the first statistical evidence supporting a refined response assessment strategy for long-term responders to immunotherapy with advanced NSCLC. A 3-month assessment interval did not significantly compromise patient survival outcomes and may also reduce annual US healthcare expenditure substantially. These findings support the adoption of a less frequent, yet equally effective, monitoring approach to make tumor surveillance more precise and cost-effective.

## Figures and Tables

**Table 1:** Baseline patient characteristics before PSM and after PSM.

Characteristics	Before PSM					After PSM				
	All Patients (*n* = 588)	2-Month Group (*n* = 229)	3-Month Group (*n* = 359)	*p*	SMD *	All Patients (*n* = 376)	2-Month Group (*n* = 188)	3-Month Group (*n* = 188)	*p*	SMD *
Gender, *n* (%)										
Female	112 (19.0)	41 (17.9)	71 (19.8)	0.648	0.048	68 (18.1)	35 (18.6)	33 (17.6)	0.893	0.028
Male	476 (81.0)	188 (82.1)	288 (80.2)			308 (81.9)	153 (81.4)	155 (82.4)		
Age, *n* (%)										
≤63 years	298 (50.7)	113 (49.3)	185 (51.5)	0.665	0.044	206 (54.8)	96 (51.1)	110 (58.5)	0.178	0.15
>63 years	290 (49.3)	116 (50.7)	174 (48.5)			170 (45.2)	92 (48.9)	78 (41.5)		
Histological type, *n* (%)										
Non-squamous	423 (71.9)	156 (68.1)	267 (74.4)	0.121	0.138	271 (72.1)	141 (75.0)	130 (69.1)	0.25	0.131
Squamous	165 (28.1)	73 (31.9)	92 (25.6)			105 (27.9)	47 (25.0)	58 (30.9)		
PFS stratification, *n* (%)										
≤median PFS	291 (49.5)	131 (57.2)	160 (44.6)	0.004	0.255	183 (48.7)	92 (48.9)	91 (48.4)	1	0.011
>median PFS	297 (50.5)	98 (42.8)	199 (55.4)			193 (51.3)	96 (51.1)	97 (51.6)		
PD-L1 TPS, *n* (%)										
<1%	37 (6.3)	11 (4.8)	26 (7.2)	0.028	0.257	8 (2.1)	6 (3.2)	2 (1.1)	0.269	0.205
1–49%	119 (20.2)	37 (16.2)	82 (22.8)			75 (19.9)	36 (19.1)	39 (20.7)		
≥50%	205 (34.9)	77 (33.6)	128 (35.7)			135 (35.9)	73 (38.8)	62 (33.0)		
Unknown	227 (38.6)	104 (45.4)	123 (34.3)			158 (42.0)	73 (38.8)	85 (45.2)		
Immunotherapy drugs, *n* (%)										
Camrelizumab	119 (20.2)	46 (20.1)	73 (20.3)	0.042	0.265	77 (20.5)	43 (22.9)	34 (18.1)	0.841	0.123
Pembrolizumab	225 (38.3)	82 (35.8)	143 (39.8)			148 (39.4)	72 (38.3)	76 (40.4)		
Tislelizumab	92 (15.6)	48 (21.0)	44 (12.3)			64 (17.0)	31 (16.5)	33 (17.6)		
Sintilimab	105 (17.9)	40 (17.5)	65 (18.1)			65 (17.3)	32 (17.0)	33 (17.6)		
Others	47 (8.0)	13 (5.7)	34 (9.5)			22 (5.9)	10 (5.3)	12 (6.4)		
Immunotherapy monotherapy, *n* (%)										
No	360 (61.2)	147 (64.2)	213 (59.3)	0.274	0.1	230 (61.2)	117 (62.2)	113 (60.1)	0.751	0.044
Yes	228 (38.8)	82 (35.8)	146 (40.7)			146 (38.8)	71 (37.8)	75 (39.9)		
Smoking history, *n* (%)										
No	175 (29.8)	61 (26.6)	114 (31.8)	0.218	0.113	115 (30.6)	51 (27.1)	64 (34.0)	0.179	0.15
Yes	413 (70.2)	168 (73.4)	245 (68.2)			261 (69.4)	137 (72.9)	124 (66.0)		
Radiotherapy history, *n* (%)										
No	299 (50.9)	81 (35.4)	218 (60.7)	<0.001	0.525	160 (42.6)	73 (38.8)	87 (46.3)	0.175	0.151
Yes	289 (49.1)	148 (64.6)	141 (39.3)			216 (57.4)	115 (61.2)	101 (53.7)		

Abbreviations: SMDs: standardized mean differences; PD-L1 TPS: Programmed Death-Ligand 1 Tumor Proportion Score; PFS: progression-free survival; PSM: propensity score matching. * SMD, standardized mean difference, was used to assess the balance of covariates after propensity score matching. An absolute SMD value of <0.1 indicates a good balance, and 0.1–0.2 is considered acceptable. For the PD-L1 TPS variable specifically, the SMD was 0.205, marginally above the conventional 0.2 threshold. We performed a sensitivity analysis by including PD-L1 TPS as an additional covariate in our Cox proportional hazards model (Table S1). Reassuringly, this adjustment resulted in only a minimal change in the hazard ratio (−3.6%), from 0.78 to 0.75, with nearly identical confidence intervals. This indicates that our primary findings are robust to the slight residual imbalance in PD-L1 expression.

**Table 2:** The univariate and multivariate analyses.

Characteristics	Univariate Analysis			Multivariate Analysis		
	HR	95% CI	*p*	HR	95% CI	*p*
Age						
≤63 years	Ref			Ref		
>63 years	1.48	1.01–2.16	0.045	2.03	1.35–3.06	0.001
Gender						
Female	Ref			-		
Male	1.23	0.75–2.02	0.416	-		
Smoking history						
No	Ref			-		
Yes	1.11	0.73–1.67	0.633	-		
Histological type						
Non-squamous	Ref			-		
Squamous	1.28	0.85–1.94	0.235	-		
PD-L1 TPS						
<1%	Ref			Ref		
1–49%	0.09	0.03–0.26	<0.001	0.29	0.10–0.84	0.022
≥50%	0.11	0.04–0.29	<0.001	0.26	0.10–0.69	0.007
Unknown	0.15	0.06–0.39	<0.001	0.33	0.13–0.85	0.021
Immunotherapy drugs						
Camrelizumab	Ref			-		
Pembrolizumab	1.62	0.92–2.87	0.095	-		
Tislelizumab	1.81	0.91–3.60	0.091	-		
Sintilimab	1.36	0.70–2.64	0.367	-		
Others	1.37	0.53–3.51	0.514	-		
Immunotherapy monotherapy						
No	Ref			Ref		
Yes	0.22	0.13–0.35	<0.001	0.24	0.14–0.40	<0.001
Response assessment						
2-month	Ref			-		
3-month	0.78	0.53–1.14	0.200	-		
PFS stratification						
≤median PFS	Ref			Ref		
>median PFS	0.11	0.07–0.17	<0.001	0.13	0.08–0.21	<0.001
Radiotherapy history						
No	Ref			-		
Yes	1.25	0.83–1.87	0.292	-		

Abbreviations: CI: confidence interval; Ref: reference; HR: hazard ratio; PD-L1 TPS: Programmed Death-Ligand 1 Tumor Proportion Score; PFS: progression-free survival.

**Table 3:** Standard charges of top cancer hospitals in the US.

Top Cancer Hospitals in the US *	CT Scan of Chest with and Without Contrast		CT Abdomen with and Without Contrast		CBC		CMP		TSH		FT4	
	Min Charge	Max Charge	Min Charge	Max Charge	Min Charge	Max Charge	Min Charge	Max Charge	Min Charge	Max Charge	Min Charge	Max Charge
University of Texas MD Anderson Cancer Center	180.34	3605.98	180.34	3817.54	5.43	86.86	8.87	649.30	14.11	254.56	7.58	138.46
Memorial Sloan Kettering Cancer Center	383.97	2604.60	383.97	2168.00	13.65	134.10	31.86	323.10	31.13	342.90	31.13	263.70
Massachusetts General Hospital	167.95	1305.68	201.74	1305.68	6.34	48.56	10.35	79.34	16.46	126.10	8.84	67.67
Mount Sinai Hospital	164.11	775.89	191.23	1042.28	65.76	78.72	137.00	164.00	109.60	131.20	NA	NA
The Hospital of the University of Pennsylvania	93.36	7221.00	93.36	7370.00	3.89	260.00	5.28	350.00	6.00	75.00	4.51	369.00
Mean	197.95	3102.63	210.13	3140.70	19.01	121.65	38.67	313.15	35.46	185.95	13.02	209.71
Min mean standard charge	USD 514.24											
Max mean standard charge	USD 7073.79											

* Of the top ten cancer hospitals (as ranked by U.S. News & World Report, https://health.usnews.com/best-hospitals/rankings/cancer, accessed on 26 July 2025), standard charge (both minimum and maximum) is available for five of them. Abbreviations: CBC: complete blood count; CMP: comprehensive metabolic panel; CT: computed tomography; FT4: free thyroxine; Min: minimum; Max: maximum; NA: not applicable; TSH: thyroid-stimulating hormone; the US: the United States.

## Data Availability

The data that support the findings of this study are available from the corresponding author, Youling Gong, upon reasonable request.

## Supplementary Materials

The following supporting information can be downloaded at: https://www.mdpi.com/article/10.3390/cancers17203312/s1, Table S1: Sensitivity Analysis; Table S2: Baseline patient characteristics of combination therapy and immunotherapy monotherapy.

## Abbreviations

The following abbreviations are used in this manuscript:

NSCLC	Non-small-cell lung cancer
PFS	Progression-free survival
OS	Overall survival
PSM	Propensity score matching
CMA	Cost minimization analysis
HR	Hazard ratio
CI	Confidence interval
SMD	Standardized mean difference
PD-1	Programmed death-1
PD-L1	Programmed death ligand 1
ICIs	Immune checkpoint inhibitors
TPS	Tumor proportion score
NCCN	National Comprehensive Cancer Network
ECOG-PS	Eastern Cooperative Oncology Group Performance Status
HIS	Health Information System
RECIST	The Response Evaluation Criteria In Solid Tumors
WHO	World Health Organization
irAEs	Immune-related adverse events
CBC	Complete blood count
CMP	Comprehensive metabolic panel
TSH	Thyroid-stimulating hormone
FT4	Free thyroxine
mOS	Median OS
IQR	Interquartile range
NR	Not reached
The US	The United States
TMB	Tumor mutational burden
SCNAs	Somatic copy number alterations
ASCO	American Society of Clinical Oncology
QALYs	Quality-adjusted life years
CUA	Cost–utility analysis

## References

[1] Bray F., Laversanne M., Sung H., Ferlay J., Siegel R.L., Soerjomataram I., Jemal A. (2024). Global Cancer Statistics 2022: GLOBOCAN Estimates of Incidence and Mortality Worldwide for 36 Cancers in 185 Countries. CA A Cancer J Clin..

[2] Sung H., Ferlay J., Siegel R.L., Laversanne M., Soerjomataram I., Jemal A., Bray F. (2021). Global Cancer Statistics 2020: GLOBOCAN Estimates of Incidence and Mortality Worldwide for 36 Cancers in 185 Countries. CA A Cancer J Clin..

[3] Cagle P.T., Allen T.C., Olsen R.J. (2013). Lung Cancer Biomarkers: Present Status and Future Developments. Arch. Pathol. Lab. Med..

[4] Molina J.R., Yang P., Cassivi S.D., Schild S.E., Adjei A.A. (2008). Non-Small Cell Lung Cancer: Epidemiology, Risk Factors, Treatment, and Survivorship. Mayo Clin. Proc..

[5] Brahmer J.R., Lee J.-S., Ciuleanu T.-E., Bernabe Caro R., Nishio M., Urban L., Audigier-Valette C., Lupinacci L., Sangha R., Pluzanski A. (2023). Five-Year Survival Outcomes with Nivolumab Plus Ipilimumab Versus Chemotherapy as First-Line Treatment for Metastatic Non–Small-Cell Lung Cancer in CheckMate 227. J. Clin. Oncol..

[6] Socinski M.A., Jotte R.M., Cappuzzo F., Orlandi F., Stroyakovskiy D., Nogami N., Rodríguez-Abreu D., Moro-Sibilot D., Thomas C.A., Barlesi F. (2018). Atezolizumab for First-Line Treatment of Metastatic Nonsquamous NSCLC. N. Engl. J. Med..

[7] Gandhi L., Rodríguez-Abreu D., Gadgeel S., Esteban E., Felip E., De Angelis F., Domine M., Clingan P., Hochmair M.J., Powell S.F. (2018). Pembrolizumab plus Chemotherapy in Metastatic Non–Small-Cell Lung Cancer. N. Engl. J. Med..

[8] De Castro G., Kudaba I., Wu Y.-L., Lopes G., Kowalski D.M., Turna H.Z., Caglevic C., Zhang L., Karaszewska B., Laktionov K.K. (2023). Five-Year Outcomes with Pembrolizumab Versus Chemotherapy as First-Line Therapy in Patients with Non–Small-Cell Lung Cancer and Programmed Death Ligand-1 Tumor Proportion Score ≥ 1% in the KEYNOTE-042 Study. J. Clin. Oncol..

[9] Tan S., Li D., Zhu X. (2020). Cancer Immunotherapy: Pros, Cons and Beyond. Biomed. Pharmacother..

[10] Huang Q., Zhang H., Hai J., Socinski M.A., Lim E., Chen H., Stebbing J. (2018). Impact of PD-L1 Expression, Driver Mutations and Clinical Characteristics on Survival after Anti-PD-1/PD-L1 Immunotherapy versus Chemotherapy in Non-Small-Cell Lung Cancer: A Meta-Analysis of Randomized Trials. OncoImmunology.

[11] Reck M., Rodríguez–Abreu D., Robinson A.G., Hui R., Csőszi T., Fülöp A., Gottfried M., Peled N., Tafreshi A., Cuffe S. (2019). Updated Analysis of KEYNOTE-024: Pembrolizumab Versus Platinum-Based Chemotherapy for Advanced Non–Small-Cell Lung Cancer with PD-L1 Tumor Proportion Score of 50% or Greater. J. Clin. Oncol..

[12] Gadgeel S., Rodríguez-Abreu D., Speranza G., Esteban E., Felip E., Dómine M., Hui R., Hochmair M.J., Clingan P., Powell S.F. (2020). Updated Analysis from KEYNOTE-189: Pembrolizumab or Placebo Plus Pemetrexed and Platinum for Previously Untreated Metastatic Nonsquamous Non–Small-Cell Lung Cancer. J. Clin. Oncol..

[13] Novello S., Kowalski D.M., Luft A., Gümüş M., Vicente D., Mazières J., Rodríguez-Cid J., Tafreshi A., Cheng Y., Lee K.H. (2023). Pembrolizumab Plus Chemotherapy in Squamous Non–Small-Cell Lung Cancer: 5-Year Update of the Phase III KEYNOTE-407 Study. J. Clin. Oncol..

[14] Migliorino M.R., Santo A., Romano G., Cortinovis D., Galetta D., Alabiso O., Cartenì G., Vari S., Fasola G., Pazzola A. (2017). Economic Burden of Patients Affected by Non-Small Cell Lung Cancer (NSCLC): The LIFE Study. J. Cancer Res. Clin. Oncol..

[15] Riely G.J., Wood D.E., Ettinger D.S., Aisner D.L., Akerley W., Bauman J.R., Bharat A., Bruno D.S., Chang J.Y., Chirieac L.R. (2024). Non–Small Cell Lung Cancer, Version 4.2024. J. Natl. Compr. Cancer Netw..

[16] Welch H.G., Dossett L.A. (2025). Routine Surveillance for Cancer Metastases—Does It Help or Harm Patients?. N. Engl. J. Med..

[17] Novello S., Barlesi F., Califano R., Cufer T., Ekman S., Levra M.G., Kerr K., Popat S., Reck M., Senan S. (2016). Metastatic Non-Small-Cell Lung Cancer: ESMO Clinical Practice Guidelines for Diagnosis, Treatment and Follow-Up. Ann. Oncol..

[18] Shi J.-F., Wang L., Wu N., Li J.-L., Hui Z.-G., Liu S.-M., Yang B.-Y., Gao S.-G., Ren J.-S., Huang H.-Y. (2019). Clinical Characteristics and Medical Service Utilization of Lung Cancer in China, 2005–2014: Overall Design and Results from a Multicenter Retrospective Epidemiologic Survey. Lung Cancer.

[19] Zhang Y.-L., Yuan J.-Q., Wang K.-F., Fu X.-H., Han X.-R., Threapleton D., Yang Z.-Y., Mao C., Tang J.-L. (2016). The Prevalence of *EGFR* Mutation in Patients with Non-Small Cell Lung Cancer: A Systematic Review and Meta-Analysis. Oncotarget.

[20] Rikova K., Guo A., Zeng Q., Possemato A., Yu J., Haack H., Nardone J., Lee K., Reeves C., Li Y. (2007). Global Survey of Phosphotyrosine Signaling Identifies Oncogenic Kinases in Lung Cancer. Cell.

[21] Zhang Q., Wu C., Ding W., Zhang Z., Qiu X., Mu D., Zhang H., Xi Y., Zhou J., Ma L. (2019). Prevalence of ROS1 Fusion in Chinese Patients with Non-small Cell Lung Cancer. Thorac. Cancer.

[22] Brahmer J.R., Lacchetti C., Schneider B.J., Atkins M.B., Brassil K.J., Caterino J.M., Chau I., Ernstoff M.S., Gardner J.M., Ginex P. (2018). Management of Immune-Related Adverse Events in Patients Treated with Immune Checkpoint Inhibitor Therapy: American Society of Clinical Oncology Clinical Practice Guideline. J. Clin. Oncol..

[23] Frigola J., Navarro A., Carbonell C., Callejo A., Iranzo P., Cedrés S., Martinez-Marti A., Pardo N., Saoudi-Gonzalez N., Martinez D. (2021). Molecular Profiling of Long-term Responders to Immune Checkpoint Inhibitors in Advanced Non-small Cell Lung Cancer. Mol. Oncol..

[24] Wang D.Y., Salem J.-E., Cohen J.V., Chandra S., Menzer C., Ye F., Zhao S., Das S., Beckermann K.E., Ha L. (2018). Fatal Toxic Effects Associated with Immune Checkpoint Inhibitors: A Systematic Review and Meta-Analysis. JAMA Oncol..

[25] Ramos-Casals M., Sisó-Almirall A. (2024). Immune-Related Adverse Events of Immune Checkpoint Inhibitors. Ann. Intern. Med..

[26] Hauptmann M., Byrnes G., Cardis E., Bernier M.-O., Blettner M., Dabin J., Engels H., Istad T.S., Johansen C., Kaijser M. (2023). Brain Cancer after Radiation Exposure from CT Examinations of Children and Young Adults: Results from the EPI-CT Cohort Study. Lancet Oncol..

[27] Yoshinaga S., Mabuchi K., Sigurdson A.J., Doody M.M., Ron E. (2004). Cancer Risks among Radiologists and Radiologic Technologists: Review of Epidemiologic Studies. Radiology.

[28] Bosch De Basea Gomez M., Thierry-Chef I., Harbron R., Hauptmann M., Byrnes G., Bernier M.-O., Le Cornet L., Dabin J., Ferro G., Istad T.S. (2023). Risk of Hematological Malignancies from CT Radiation Exposure in Children, Adolescents and Young Adults. Nat. Med..

[29] Brenner D.J., Hall E.J. (2007). Computed Tomography—An Increasing Source of Radiation Exposure. N. Engl. J. Med..

[30] Brenner D.J., Doll R., Goodhead D.T., Hall E.J., Land C.E., Little J.B., Lubin J.H., Preston D.L., Preston R.J., Puskin J.S. (2003). Cancer Risks Attributable to Low Doses of Ionizing Radiation: Assessing What We Really Know. Proc. Natl. Acad. Sci. USA.

[31] Wood R., Taylor-Stokes G. (2019). Cost Burden Associated with Advanced Non-Small Cell Lung Cancer in Europe and Influence of Disease Stage. BMC Cancer.

[32] Longo C.J., Fitch M.I., Loree J.M., Carlson L.E., Turner D., Cheung W.Y., Gopaul D., Ellis J., Ringash J., Mathews M. (2021). Patient and Family Financial Burden Associated with Cancer Treatment in Canada: A National Study. Support. Care Cancer.

