# Supplementary material for: Impact of Response Assessment Intervals on Survival and Economic Burden in Long-Term Responders to Immunotherapy for Advanced Non-Small-Cell Lung Cancer
Source: Cancers (Basel). 2025 Oct 14;17(20):3312. doi: 10.3390/cancers17203312 (PMC12563779; doi:10.3390/cancers17203312)
Supplement: Supplementary file 1 [file cancers-17-03312-s001.zip › cancers-3899474-supplementary.pdf]

Supplementary Materials

Supplementary Table S1. Sensitivity Analysis

Model	HR	95% CI	*p*	Change in HR
Primary Analysis	0.78	0.53-1.14	0.2	Ref
Sensitivity Analysis (Adjusted for PD-L1)	0.75	0.51-1.10	0.14	-3.60%

Abbreviations: CI: Confidence Interval; Ref: Reference; HR: Hazard Ratio; PD-L1: Programmed Death-Ligand 1;

Supplementary Table S2. Baseline patient characteristics of combination therapy and immunotherapy monotherapy

**Characteristics**	**Combination therapy (n=230)**	**Immunotherapy monotherapy (n=146)**	***p***
Gender, n (%)			0.804
Female	43 (18.7)	25 (17.1)	
Male	187 (81.3)	121 (82.9)	
Age, n (%)			0.001
≤ 63 years	142 (61.7)	64 (43.8)	
> 63 years	88 (38.3)	82 (56.2)	
Histological type, n (%)			0.177
Non-squamous	172 (74.8)	99 (67.8)	
Squamous	58 (25.2)	47 (32.2)	
PFS stratification, n (%)			<0.001
≤ median PFS	133 (57.8)	50 (34.2)	
> median PFS	97 (42.2)	96 (65.8)	
PD-L1 TPS, n (%)			0.042
< 1%	7 (3.0)	1 (0.7)	
1-49%	48 (20.9)	27 (18.5)	
	71 (30.9)	64 (43.8)	

≥ 50%	104 (45.2)	54 (37.0)	
Unknown			
Immunotherapy drugs, n (%)			0.006
Camrelizumab	51 (22.2)	26 (17.8)	
Pembrolizumab	89 (38.7)	59 (40.4)	
Tislelizumab	49 (21.3)	15 (10.3)	
Sintilimab	30 (13.0)	35 (24.0)	
Others	11 (4.8)	11 (7.5)	
Response assessment strategy, n (%)			0.751
2-month group	117 (50.9)	71 (48.6)	
3-month group	113 (49.1)	75 (51.4)	
Smoking history, n (%)			0.237
No	76 (33.0)	39 (26.7)	
Yes	154 (67.0)	107 (73.3)	
Radiotherapy history, n (%)			0.574
No	101 (43.9)	59 (40.4)	
Yes	129 (56.1)	87 (59.6)	

Abbreviations: PD-L1 TPS: Programmed Death-Ligand 1 Tumor Proportion Score; PFS: Progression-Free Survival;
